# Atrificial Intelligence, uses, AI in Arabic and Obstacles using AI in Jordan

**DOI:** 10.1192/j.eurpsy.2026.11541

**Published:** 2026-07-31

**Authors:** S. F. Abu Dayeh, Q. H. Al-Rimawi

**Affiliations:** Psychiatry, MOH, Amman, Jordan

## Abstract

**Introduction:**

This study discusses the major uses of AI in our lives and the aspects which can develop using AI, as these high technology tools have advantages and disadvantages that are also discussed in this study. Major questions using a survey questionnaire were answered by our randomly chosen sample in Jordan, which are 103, and then categorising them according to the age and gender. According to the results AI tools in arabic are not that much available and that is the obstacle that people have.

**Objectives:**

The Questions of the Study:

- Are there any differences in the age and gender in using AI tools ?
- What are the languages preferred using AI ?
- What are the reasons of using AI ?
- What are the obstacles of using AI in arabic ?

**Methods:**

The study was done using a survey questionnare, the sample is 103 people, chosen randomly from Jordan, categorised according to the age and gender and after obtaining the informed consent required. ANOVA was used.

**Results:**

1. Level of using AI among the participants was Moderate with a mean of 0.48.
2. No statically significant differences in using AI due to age and gender (F= 0.384 and 0.288, Sig.>0.05).
3. The language is preferred in using AI is both Arabic and English with a percentage of 44.7%. followed by using it in English 25.2%. After it came using it Arabic with a percentage of 22.3%. Finally, other languages came with a percentage of 7.8%.
4. The most reason of using AI is normal life with a percentage of 41.7%. followed by using it for Work 30.1%. After it came using it for Games and fun with a percentage of 16.5%. Finally, not applicable has a percentage of 11.7%
5. The most obstacles of using AI in Arabic language was “ Not that much available in Arabic” with a percentage of 28.2%. followed by “Unnecessary” and “Difficult to use” with 25.2%. After it came “Expensive to get” with a percentage of 15.5%. Finally, Weak internet connection came with a percentage of 5.8%.

**Conclusions:**

AI is starting to be neceessary to use in our lives that’s why more training programs for people must be done, in order to use these tools safely around the world. Cybersecurity programs should also be included in training programs, to prevent any invasion of any person’s privacy. Arabic AI tools should contain a wider structured branched algorithms, datasets, more applications and programs to be easier to get and to use.

**Disclosure of Interest:**

None Declared

